# Mitotic Diversity in Homeostatic Human Interfollicular Epidermis

**DOI:** 10.3390/ijms17020167

**Published:** 2016-01-28

**Authors:** Katharina Nöske, Hans-Jürgen Stark, Leonard Nevaril, Manuel Berning, Lutz Langbein, Ashish Goyal, Sven Diederichs, Petra Boukamp

**Affiliations:** 1 Department of Genetics of Skin Carcinogenesis, German Cancer Research Center (DKFZ), Heidelberg 69120, Germany; katharinanoeske@gmx.de (K.N.); hj.stark@dkfz.de (H.-J.S.); L.Nevaril@dkfz.de (L.N.); manuel.berning@googlemail.com (M.B.); L.Langbein@dkfz.de (L.L.); 2 Department of RNA Biology and Cancer, German Cancer Research Center (DKFZ), Heidelberg 69120, Germany; A.Goyal@dkfz.de (A.G.); S.Diederichs@dkfz.de (S.D.); 3 IUF–Leibniz Research Institute for Environmental Medicine, Düsseldorf 40225, Germany; 4 Division of Cancer Research, Department of Thoracic Surgery, Medical Center, University of Freiburg-Faculty of Medicine, University of Freiburg, Breisacher Str. 115, Freiburg 79106, Germany; 5 German Cancer Consortium (DKTK), Freiburg 79106, Germany; 6 Institute of Pathology, University Hospital Heidelberg, Im Neuenheimer Feld 224, Heidelberg 69120, Germany

**Keywords:** human epidermis, suprabasal mitosis, Numb, asymmetric mitosis, long-term organotypic culture model, epidermal differentiation

## Abstract

Despite decades of skin research, regulation of proliferation and homeostasis in human epidermis is still insufficiently understood. To address the role of mitoses in tissue regulation, we utilized human long-term skin equivalents and systematically assessed mitoses during early epidermal development and long-term epidermal regeneration. We now demonstrate four different orientations: (1) horizontal, *i.e*., parallel to the basement membrane (BM) and suggestive of symmetric divisions; (2) oblique with an angle of 45°–70°; or (3) perpendicular, suggestive of asymmetric division. In addition, we demonstrate a fourth substantial fraction of suprabasal mitoses, many of which are committed to differentiation (Keratin K10-positive). As verified also for normal human skin, this spatial mitotic organization is part of the regulatory program of human epidermal tissue homeostasis. As a potential marker for asymmetric division, we investigated for Numb and found that it was evenly spread in almost all undifferentiated keratinocytes, but indeed asymmetrically distributed in some mitoses and particularly frequent under differentiation-repressing low-calcium conditions. Numb deletion (stable knockdown by CRISPR/Cas9), however, did not affect proliferation, neither in a three-day follow up study by life cell imaging nor during a 14-day culture period, suggesting that Numb is not essential for the general control of keratinocyte division.

## 1. Introduction

Human epidermis is a stratified epithelium that follows a tightly regulated proliferation and differentiation program with a delicate interplay of stem cell self-renewal and differentiation in order to maintain tissue homeostasis. While the stem cell populations and their hierarchical order are well defined in murine epidermis and particularly in the murine hair follicle [1,2,3,4,5], less is known about human epidermis. Reliable molecular markers are lacking that could help to identify a stem cell population and, thus, to follow stem cell fate during development and tissue homeostasis of adult human interfollicular epidermis (IFE). The prevailing model of stem cell hierarchy is also controversially discussed [6]. As an acknowledged paradigm in homeostatic skin, basal epidermal cells divide either “symmetrically”, *i.e*., horizontally/in parallel to the basement membrane (BM), in order to replenish the basal precursor cell compartment or “asymmetrically” with the mitotic spindle in perpendicular orientation to the BM in order to produce progeny, initiating the differentiation program [7]. This paradigm was established from studies conducted on the IFE of mouse skin. However, distinct differences are evident between mouse and human epidermis with respect to stratification and thickness, thereby demonstrating an altered spatial regulation, not to mention the different hair follicle densities [8,9]. These differences may suggest some discrepancies in the morphogenetic regulation of human *versus* mouse epidermis, which may also include the regulation of mitotic activity.

In order to analyze the chronological/temporal and spatial regulation of mitoses in human IFE, we used our long-term skin equivalents (SEs) [10,11] which allowed us to establish the mitotic profile during epidermal establishment, *i.e*., the phase of wound healing-like regeneration, as well as during long-term regeneration, *i.e*., in tissue homeostasis. We now describe three different types of mitotic orientation in the basal layer and additionally a fraction of horizontal and oblique mitoses localized in suprabasal layers, *i.e*., in the differentiation compartment.

Orientation of the mitotic spindle is supposedly associated with symmetric and asymmetric division. Several markers have been proposed to be involved in orienting the mitotic spindle perpendicularly. Most prominent is the PAR complex with its associated proteins [12,13,14]. Additional proteins with regulatory functions are involved. Among those are members of the Notch-Numb axis. In many tissues, including the epidermis of the skin, the Notch pathway mediates lateral inhibition and thereby governs cell fate determination. It is suggested that increased expression of Notch ligands in one cell causes Notch signalling in its neighbouring cells which then will cause an alternative fate [15]. During this process modulators of Notch signalling, such as Numb, get asymmetrically localized after mitosis, staying enriched in the parental cell that remains in contact with the basement membrane while being strongly reduced in the daughter cell moving upwards into the suprabasal layer. (see for reviews [16,17]). Furthermore, it was shown that besides functioning in progenitor cell fate determination and early development, Numb directly exerts controlling functions in the cell cycle. Targeted knockdown of Numb expression caused a G(2)-M arrest and thus reduced cell growth in human melanoma cells [18]. Finally, a recent study suggested that Numb is involved in maintaining the “the undifferentiated proliferating stem cell pool in the epithelial basal layer” [19] thus making Numb an excellent candidate for defining asymmetric division and prompting us to study its role in the mitotic regulation of human skin keratinocytes. Here, we assess the profile of Numb distribution during mitosis in cultured keratinocytes and show that CRISPR/Cas9-dependent knockdown of Numb does not affect proliferation. These data exclude an essential role of Numb in human skin keratinocytes for traversing mitosis but rather support its function in the control of mitotic orientation.

## 2. Results and Discussion

### 2.1. Four Different Orientations Characterize Mitoses in Human Interfollicular Epidermis

**Figure 1:**
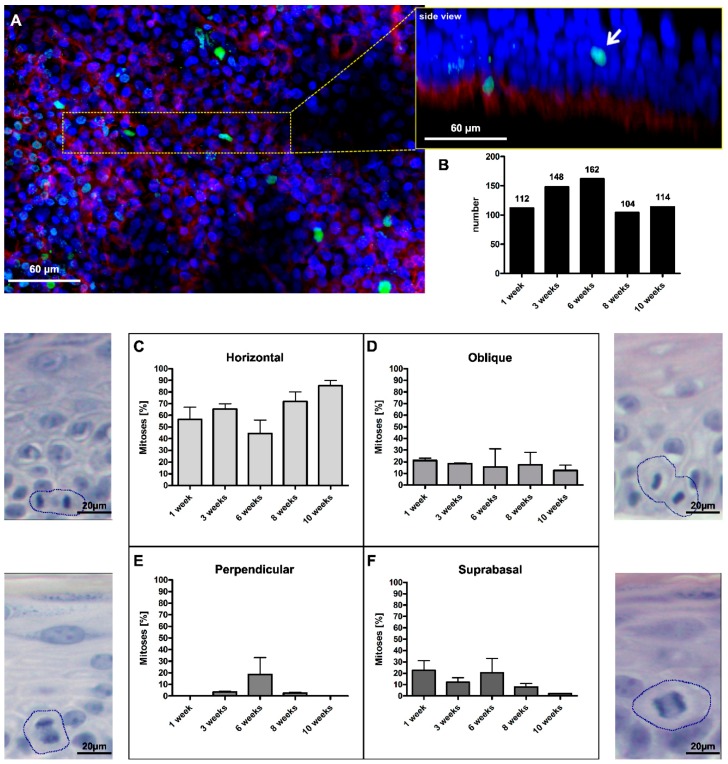
Four different types of mitoses were found in the human skin equivalent. (**A**) Whole mounts of the SE were stained with anti-integrin α6 (red) and anti-H3S10ph (green) and confocally analyzed to visualize the localization and angle of mitotic events. The insert represents the 3D reconstruction of the magnified tissue piece, seen from the side, the white arrow indicates a suprabasal mitotic cell; (**B**) In total, 640 mitotic events were counted at culture time points ranging from 1–10 weeks; (**C**) 40%–80% of mitoses occurred horizontally to the BM; (**D**) 10%–20% of cells divided at an oblique angles; (**E**) up to 20% of divisions were perpendicularly oriented to the BM; and (**F**) up to 20% of divisions occurred suprabasally. Error bars in (**C**–**F**): standard error of the mean (SEM) of three individual culture experiments. The accompanying micrographs in (**C**–**F**) illustrate the histological appearance in H&E-stained sections. Circles mark dividing cells.

To establish a temporal and spatial profile of mitotic events in human epidermis, we utilized fibroblast-derived matrix-based skin equivalents (SE) with normal human epidermal keratinocytes (NHEKs) and dermal fibroblasts. In this experimental set up, NHEKs establish an epidermis that is morphologically and functionally almost undistinguishable from normal human epidermis *in situ* [10,20]. After an initial phase of wound-type regeneration, *i.e.*, hyperproliferation with the consequence of hyperplasia, the cultures enter into a state of tissue homeostasis with reduced proliferation and an apparent reduction of cell layers. In this state, they maintain epidermal integrity and regeneration for several months (Figure S1A). This model was used to pursue the fate of mitoses throughout epidermal establishment and long-term regeneration in order to record which mitotic orientation would participate in stratification and homeostatic regeneration of the epidermis. In a systematic screen, we identified three distinct orientations of mitoses in the basal layer of the epidermis: (1) horizontal mitoses, *i.e*., arranged in parallel to the BM with both daughter cells remaining in contact with the BM, classically considered as “symmetric division”; (2) mitoses with an oblique angle, mostly between 45° and 70°, to the BM; and (3) mitoses arranged perpendicularly to the BM, placing one daughter cell into the suprabasal layer and being considered as “asymmetric division”. As a fourth type, we identified mitoses in the first suprabasal layer dividing in a horizontal or oblique manner. Importantly, these suprabasal mitoses were not only present during early establishment of the epidermis when the wound-like epidermis is hyperplastic, but also in later stages of homeostatic regeneration when the epidermis was reduced to 4–5 cell layers (Figure 1).

To quantify the number of mitotic events, we screened tissue sections from SEs cultured for four days up to five months to monitor the diversity of mitotic events over an extensive time span (Figure S1B). However, and despite screening many sections, the number of detectable mitoses remained low. As an alternative technical approach, we prepared whole mounts from SEs cultured from 1 up to 10 weeks. In these whole mounts, the basal layer of the epidermis was highlighted by staining for the hemidesmosomal α6 integrin, and mitoses were visualized by staining with the spindle-associated protein TPX2 [21]. By laser scan microscopy and 3D reconstruction of the confocal z-stacks, the localization and orientation of the mitotic events could be determined in the tissue context in a larger tissue piece (Figure 1A). In three independent experiments, we identified 640 mitotic events (Figure 1B), which well recapitulated the diversity already seen in tissue sections (Figure S1C–F).

The most prominent type was horizontal mitosis with 40%–80% (Figure 1C). Horizontal mitoses were particularly dominant (90%) at later time points when the number of vital cell layers was reduced and the epidermis had approached homeostatic regeneration. The second most frequent type of mitosis (around 20%) exhibited an oblique division angle (Figure 1D). This type of mitosis appeared with similar frequency at all time points. Comparable occurrences of mitoses with an oblique orientation were found in basal cells of mouse embryonic (E16.5) skin [22], suggesting that also this orientation is a common feature of the stratifying epidermis. The number of perpendicular mitoses, *i.e*., potential asymmetric divisions, on the other hand, was generally low (Figure 1E). Only at Week 6 was a more substantial rate (~20%) of perpendicular mitoses identified. Finally, we found a substantial proportion of suprabasal mitoses. Although more frequent at the early time points (up to 20%), we identified suprabasal proliferation also upon long-term regeneration, *i.e*., in a phase of tissue homeostasis (Figure 1F). So far, suprabasal mitoses were commonly ascribed only to the early embryonic stages of skin development, wound situations or skin diseases [7,23,24]. Accordingly, we asked whether reactivation of the homeostatic epidermis would preferentially stimulate suprabasal proliferation. Keratolysis was performed on the epidermis of 10-week-old SE by urea treatment. Two days after peeling of the stratum corneum, proliferation was massively increased in the already hyperplastic epidermis (Figure S2A,B). Quantification of basal *versus* suprabasal Ki67-positive nuclei supported an about 35% suprabasal fraction in this early stage of keratolysis-induced hyperproliferation. Five days later, proliferation was largely normalized, leaving a 10%–20% fraction of suprabasal Ki67-positive cells (Figure S2C).

Together, tissue homeostasis in human epidermis is maintained by a fine-tuned interplay between horizontal and oblique mitoses occurring in the regenerative basal layer, as well as suprabasal mitoses of already committed differentiating cells, thus making suprabasal mitoses an integral part of this regulatory program. Disrupting tissue homeostasis by, e.g., keratolysis induces a massive burst of proliferation encompassing both the basal and suprabasal layer. This again argues for a defined role of suprabasal mitoses in rapid wound response. Perpendicular mitoses, on the other hand, appear only rarely. Whether they are restricted to stem cell divisions has yet to be determined.

### 2.2. Suprabasal Mitosis as a Part of Tissue Homeostasis

**Figure 2:**
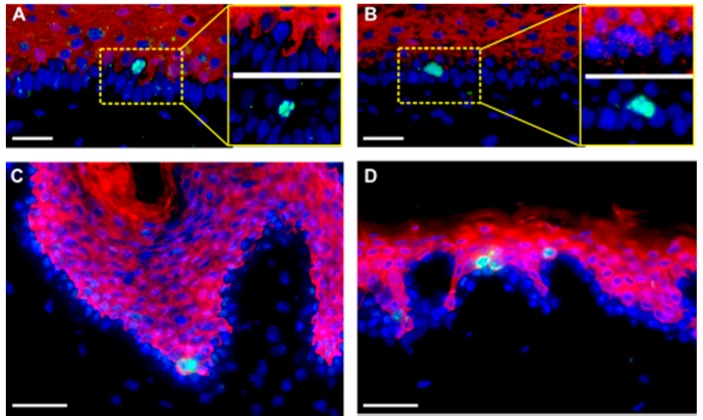
Suprabasal division is not an uncommon feature of the human homeostatic epidermis in SE and human skin *in situ*. (**A**,**B**) Sections of the SE, five weeks old and (**C**,**D**) normal human skin were stained with anti-H3Sph10 (green) to visualize mitotic cells and anti-K10 (red) to show differentiating cells. Suprabasally-dividing cells were frequently found in both systems. Error bars **A**–**D**: 100 µm.

**Figure 3:**
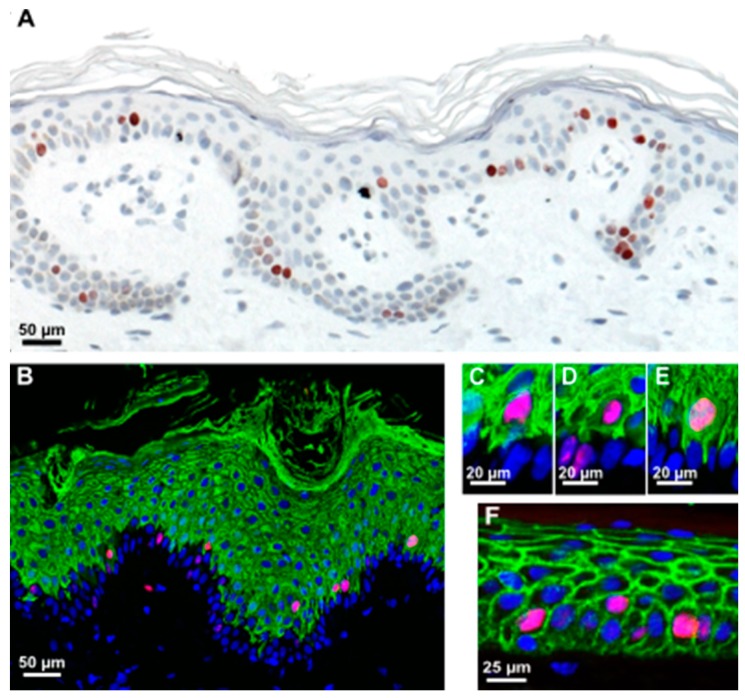
Most suprabasally-dividing cells are already committed to differentiation. (**A**) Immunohistochemistry of skin sections revealing Ki67-positive mitotic cells in both the basal and suprabasal cell layers in normal human skin; (**B**) Many mitotic cells (red, stained with anti-Ki67) are positive for the early differentiation marker K10 (green) in normal human skin; (**C**–**E**) Insets of (**B**) reveal that many of these suprabasally-dividing cells often retain cytoplasmic connections, reaching the BM, while few are really separated from the BM (**D**), as indicated by a complete lining with E-cadherin (**F**).

Having identified a substantial fraction of suprabasal mitoses even during long-term regeneration in homeostatic epidermis in the SE (Figure 1 and Figure 2A), we asked whether this was specific for our experimental model or would reflect a common regulatory feature also for normal human skin *in situ.* Therefore, we screened sections of various skin samples for the specific mitosis marker phospho-histone H3 (H3S10ph) (Figure 2) or the proliferation marker Ki67 (Figure 3) [25,26,27]. As exemplified in Figure 3, Ki67-positive cells were present in, but not restricted to, the K14-positive stratum basale, as were H3S10ph-positive cells, which mostly occurred in the K10-positive stratum spinosum. Suprabasal mitotic cells were seen in rete-rich, as well as more stretched rete-poor epidermis. Quantitation of Ki67 in different skin samples demonstrated an actual majority of suprabasal Ki67-positive nuclei and, despite the limited number of samples, showed a trend toward a decrease of this suprabasal fraction with age (Figure S3A). Interestingly, in plantar skin, the number of suprabasal Ki67-positive nuclei exceeded the number of basal-positive nuclei by 2–3-fold, while this ratio in scalp and trunk skin was about 1.5-fold (Figure S3) Thus, suprabasal proliferation was clearly not an experimental artefact, but recapitulated a common condition in human homeostatic IFE.

Two possible mechanisms could account for these suprabasal mitoses: keratinocytes may detach from the BM during division due to spatial restrictions in the basal cell layer and reinsert in the basal layer after division, as was observed in the branching ureteric bud epithelium [28]; alternatively, they may be genuine suprabasal, early differentiating cells. To address this, we investigated the differentiation status and localization within the tissue in a more precise manner. Sections of the SE at different time points and healthy human skin were investigated for the proliferation markers Ki67 or H3S10ph and keratin K10 as a marker for early epidermal differentiation. We found that most of these suprabasal cells were K10-positive (Figure 2 and Figure 3). Interestingly, only a few of these cells were completely separated from the BM (Figure 3D). Those are the ones fully encased by E-cadherin (Figure 3F). Most of them exhibited cytoplasmic extensions between the basal cells reaching down to the BM and suggesting a physical connection with the BM (Figure 3C,E). It is noteworthy that we also identified single basal keratinocytes, which were weakly stained for K10. This again suggests that induction of differentiation is not exclusively regulated by asymmetric division and that there is no strict spatial separation of proliferation and differentiation.

Together, we now show that suprabasal proliferation is a common feature of normal homeostatic human skin. Interestingly, plantar skin is characterized by a particularly high amount of suprabasal Ki67-positive cells, which reflects the physiologic hyperplasia of this specific epidermal region with its increased cornification. Furthermore, proliferation of suprabasal keratinocytes in the adult human epidermis is compatible with early epidermal differentiation, as confirmed by their K10 expression. Thus, mechanistic models based on the assumption that keratinocytes strictly discriminate between proliferation and differentiation are not realistic. Whether this points to a higher regulatory flexibility or a general difference in the spatial regulation of proliferation in human *versus* mouse epidermis cannot be decided, yet. It also remains to be elucidated if there is a functional connection between the physical contact of proliferating suprabasal cells to the BM and their control of proliferation and induction of differentiation.

### 2.3. Numb Is Asymmetrically Distributed during the Division of Undifferentiated Keratinocytes

As demonstrated above, tissue homeostasis of human IFE is maintained by spatially- and topographically-different types of mitoses. The difference in orientation may reflect different regulatory conditions specifically designed to discriminate asymmetric *versus* symmetric cell division. As a prime candidate, we assessed the role of Numb for asymmetric division in human keratinocytes.

**Figure 4:**
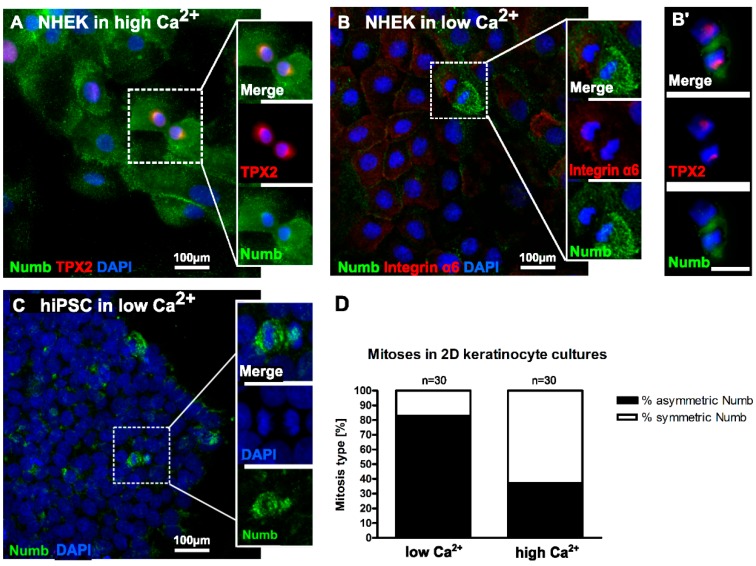
Differential distribution of Numb during mitoses depends on the differentiation state of NHEK. (**A**) In conventional culture in high Ca^2+^ medium, Numb is rather evenly distributed throughout the cytoplasm with slight accumulation at the plasma membrane. Furthermore, during most mitoses, Numb is evenly distributed; (**B**,**B’**) Under differentiation-inhibiting conditions in low calcium, Numb frequently displays an unequal distribution to the daughter cells during mitoses; (**C**) hiPSC in low Ca^2+^ medium showed an overall reduced Numb expression. However, upon division, Numb was prominent, and the protein became unequally distributed between the daughter cells; (**D**) Quantification of mitoses with symmetric and asymmetric Numb distribution in NHEK cultures in low and high calcium medium. TPX2: Targeting protein for Xklp2; DAPI: 4′,6-Diamidin-2-phenylindol; Scale bars in **B**’ and all insets 50 µm.

Investigating cultured NHEK for Numb expression, we found Numb protein to be distributed equally throughout the cytoplasm with some concentration at the plasma membrane. During mitosis, Numb expression was preserved. Upon closer inspection, however, we found two modes of distribution with (i) both daughter cells containing equal amounts of Numb and, thus, suggestive of a symmetric division (Figure 4A) and (ii) an unequal distribution of Numb in the two daughter cells during mitosis, thus indicative of asymmetric division (Figure 4B). Differential distribution of Numb expression was particularly prominent in mitoses of human-induced pluripotent stem cell (hiPSC) cultures (Figure 4C). Since those cells have been largely withdrawn from differentiation by reprogramming, the observed Numb distribution confirms that the persistence of an undifferentiated state tends to involve asymmetric cell division [29].

To address potential regulatory consequences, we next compared NHEK grown under different calcium conditions. Importantly, the culture conditions seemed to determine the frequency of the respective type of mitoses (Figure 4D). Quantification of mitotic events demonstrated a frequency of 80% of mitoses with asymmetric Numb distribution *versus* 20% symmetric distribution in low calcium medium, while the relation reverted in high calcium medium, with 30% asymmetric *versus* 70% symmetric Numb distribution. This could suggest that the regulatory program determining symmetric *versus* asymmetric distribution, and with that symmetric *versus* asymmetric division, is influenced by the level of calcium.

In normal human epidermis, as well as the epidermis in the SE, Numb was similarly expressed in the basal and suprabasal layers. Interestingly, accumulation was prominent at the basal pole of some basal cells, which appeared in clusters rather than as individually-distributed cells (Figure S4). Individual mitotic cells could not be identified in these immunofluorescence analyses, thus leaving open a role for Numb in asymmetric division in the epidermis *in situ*.

### 2.4. CRISPR/Cas9 Knockdown of Numb in Human Keratinocytes

**Figure 5:**
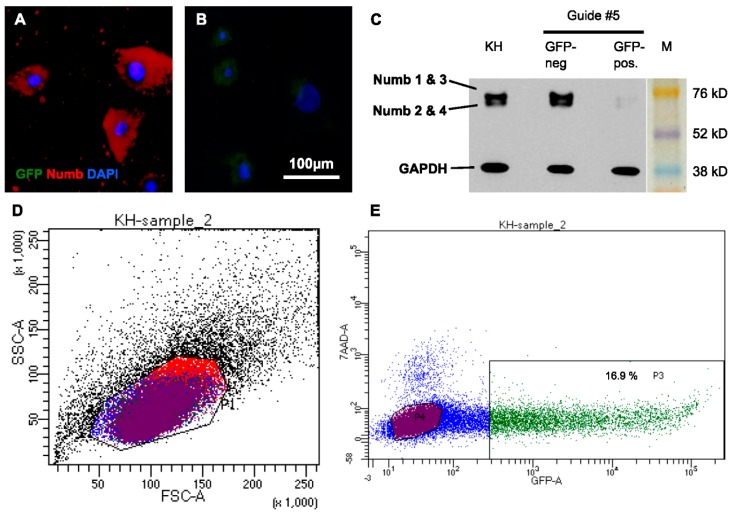
Numb is stably knocked down in human keratinocytes by CRISPR/Cas9. NHEK cells were transfected with plasmids carrying Cas9 and different gRNAs. Due to co-expression of GFP, successfully transfected cells were collected by FACS and reseeded. (**A**) After two weeks of cultivation, Numb protein was abundantly detected in sorted, GFP-negative cells, while (**B**) no protein was detected in the GFP-positive (knockdown) fraction; (**C**) The efficient knockdown of Numb in all of its isoforms was confirmed by Western Blot analysis of lysates of these cells and skin (KH) for comparison; (**D**) FACS revealed that the transfected cells were largely viable; (**E**) Between 12% and 20% of the transfected cells had taken up the CRISPR/Cas9-construct and were GFP positive in seven individual experiments. Gating: All cells (black), Live, 7AAD-negative cells (blue), GFP-neagtive (purple), GFP-positive (green), cells gated out in FFS/SSC (red). GFP: Green fluorescent protein; DAPI: 4′,6-Diamidin-2-phenylindol; KH: human keratinocytes; M: Marker; GAPDH: Glyceraldehyde-3-Phosphate Dehydrogenase; FSC: Forward scatter; SSC: Side scatter; 7AAD: 7-aminoactinomycin D.

To further address the function of Numb during mitosis, we aimed at eliminating Numb by performing a CRISPR/Cas9 knockdown in the NHEK. To knockdown Numb, five different guide RNAs were created and cloned into a Cas9 plasmid with an additional GFP reporter protein sequence, which allowed for positive selection of the successfully-transfected cells by FACS. The protein knockdown was efficient with all of the guide RNAs and highly reproducible, as demonstrated in seven independent experiments (Figure 5).

A recent study showed that Numb is required for the progression of normal (symmetric) mitosis [18]. Interestingly, deletion of Numb in the NHEK did not impair proliferation. Two weeks after seeding, the sorted cells showed slightly decreased cell numbers in comparison to untreated cells, but the Numb-deficient cells proliferated at a similar rate as the sorted GFP-negative Numb-positive cells, thus demonstrating that Numb depletion did not abrogate proliferation in the human keratinocytes.

**Figure 6:**
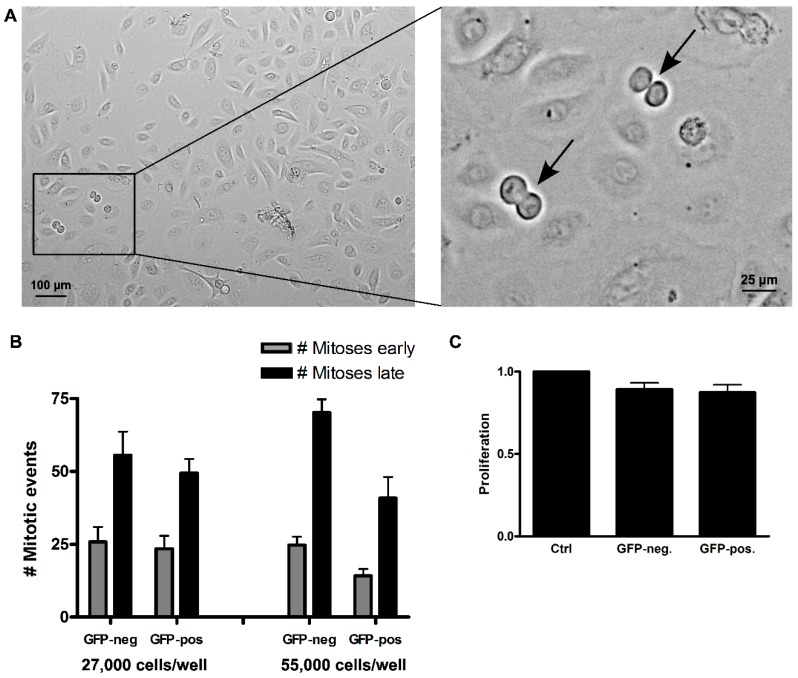
Effect of Numb knockdown on keratinocyte proliferation evaluated by live imaging. Equal cell numbers of FAC-sorted GFP-negative and GFP-positive cells were seeded and monitored for three consecutive days. (**A**) Representative example of one time point, showing two mitotic events (arrows); (**B**) Total mitotic events were counted over the first half (“early”, grey bars) and the second half of the time course (“late”, black bars). Comparison of mitotic numbers demonstrated a doubling for both populations at a seeding density of 27,000 cells, with roughly the same number of mitotic events at a seeding density of 55,000 cell/well; (**C**) Cell numbers were assessed by Crystal violet assay after two weeks of culture. Error bars: SEM of four replicates.

While this contradicted the recent finding that Numb knockdown caused reduced growth in melanoma cells by inducing a G2/M arrest [18], we were still interested in whether cells lacking Numb would progress through mitosis unhampered. We therefore followed Numb knockdown keratinocytes by live cell imaging for three consecutive days (Figure 6). The cells were investigated at two cell concentrations (27,000 and 55,000 cells/well in 24-well plates). Quantification of mitoses occurring during the first 36 h (“early”) *versus* the second 36 h (“late”) demonstrated that the number of mitoses doubled in the second 36-h period in all cultures of low cell density and about tripled in the cultures of high cell density, thus arguing for a preserved density dependency on the proliferative activity. The sorted cells exhibited a similar number of mitoses in the first half, irrespective of the initially plated cell number, thus demonstrating only a minor effect of the FACS procedure and, in particular, no sign of cell cycle arrest upon Numb-depletion (Supplementary Video). Furthermore, we recently reported an example of “impaired mitoses” for NoRC/Tip5 knockdown cells showing severe disturbance and delay of cell division because of abortive chromosome alignment [30]. In the keratinocytes with Numb knockdown, we could not constitute a mitotic phenotype. Together, this argues that Numb depletion does not impair proliferation and more specifically does not seem to interfere with cell cycling and passing through mitosis. Whether this is a keratinocyte-specific feature has to remain open for now.

In conclusion, early embryogenic development of mouse epidermis is characterised by mostly symmetric division in the still single-layered epithelium. However, upon induction of stratification (E12.5) most mitotic cells had a perpendicular orientation (~80%), generally described as asymmetric division [31]. This dominance of perpendicular mitoses was maintained also in the adult epidermis [22] or was reduced to about 35% [32]. For mouse tail epidermis, on the other hand, quite a different profile was described, with mostly parallel and oblique mitoses and only few (3%) perpendicular mitoses [33]. For human epidermis, we now present a profile that shows a similar variety of mitotic orientations as mouse tail epidermis, and that, in addition, is characterised by a remarkable amount of suprabasal mitoses. Most importantly, and despite prominent stratification, horizontal (parallel) mitoses predominated in homeostatic adult human epidermis (60%–80%) while the number of perpendicular mitoses was generally low (around 10%). Moreover, we found a substantial number of oblique mitoses. Poulsen and Lechler [34], on the other hand, suggested that spindle orientation is determined in late anaphase and, accordingly, the entire spectrum of spindle angles is displayed during late metaphase/anaphase with two predominating classes, parallel (0°–10°) and perpendicular (70°–90°) mitoses. However, in human epidermis, and as also described for mouse tail epidermis [33], anaphase alignment appears less strict leaving a constant fraction (~20%) of oblique mitotic events (>45°–<70°). Whether these are functionally identical to perpendicular mitoses and, thus, represent asymmetric divisions that contribute to epidermal stratification and differentiation remains to be seen.

Finally, and most importantly, we found numerous (10%–75%) suprabasal mitoses. This was not specific for epidermal regeneration in our skin model, but characteristic also for adult skin *in situ*. In mouse skin, only a few divisions were described to occur suprabasally during a short time window of early embryonic epidermal stratification [35]. In human skin, suprabasal epidermal proliferation was so far only associated with wound healing and disease, e.g., psoriasis [36]. It is noteworthy that the frequency of suprabasal mitoses varied strongly within a given skin, as well as between skin samples from different donors, which argues against a “fixed” ratio of basal *versus* suprabasal mitoses. Irrespective of that, however, suprabasal proliferation appears as a prominent regulatory event in human homeostatic skin. Both in mouse and in human skin, these suprabasal cells were scheduled for differentiation as they expressed the early differentiation markers Keratins K1 or K10. This clearly contradicts the general view “once suprabasal, cells stop dividing and enter a differentiation program to form the barrier” [37]. Instead of being mutually exclusive, it demonstrates that early differentiation is compatible with proliferation. This may provide human epidermis, even in the homeostatic state, with higher flexibility to react to changing demands. Furthermore, suprabasal division may offer a certain advantage to steadily expand the differentiation compartment and, thus, to provide selective protection for the basal cells. In this context, it is noteworthy that the early state of differentiation is not only compatible with proliferation, but that, for example, inactivation of telomerase activity is still reversible [31]. Thus, it appears that in the initial state of differentiation, keratinocytes can be fully reactivated.

Recent elegant work has linked spindle orientation and cell fate with differential activation of the Notch pathway (for a review, see [38]). In its function as a specific inhibitor for Notch signaling, Numb has gained considerable interest, and it was shown that “by partitioning differentially between the two daughter cells, Numb controls their fate” [39,40,41,42]. *In vivo* Numb expression was already shown for normal human mammary gland [43] and the luminal epithelial layer of Keratin K8+ cells, while its expression was absent or low in the basal/myoepithelial layer of p63+ cells of the murine mammary gland. Interestingly, rare basal p63+ cells, when occasionally dividing, showed asymmetric distribution of Numb to one of the progeny, while in dividing luminal cells, Numb segregation was predominantly symmetric [44]. Furthermore, for murine epidermis, Numb expression was demonstrated [32,33]. In agreement with those studies, we here show clusters of Numb-positive basal cells in human epidermis. In cell culture, on the other hand, almost all keratinocytes are Numb-positive, and we identified mitoses with symmetric, as well as asymmetric distribution of Numb to the daughter cells. Interestingly, the external calcium concentration, known to influence the differentiation of the keratinocytes, caused a shift from 80% of asymmetric distribution at low Ca^2+^ to 30% at high Ca^2+^, suggesting that calcium is involved in Numb-dependent fate decision. Together with the recently-described regulation of Ca^2+^ influx and signaling pathway via the TRPV6 (calcium channel)-Numb1 interaction [45], these data thus prompt us to propose a role for Ca^2+^ in the regulation of symmetric *versus* asymmetric Numb distribution. Whether also *in vivo*, the distribution of Numb is Ca^2+^-dependent, and whether this Ca^2+^ dependency defines asymmetric mitoses or even specifically stem cell mitoses awaits further investigations. In marked contrast to melanoma cells [18], however, knockdown of Numb in the human normal keratinocytes did not result in growth arrest. Proliferation remained largely unaffected, as did mitotic progression. Still, the transit into differentiation may be accelerated when Numb is depleted, suggesting that Numb regulation is part of the fate decision required for normal epidermal stratification and differentiation.

## 3. Materials and Methods

### 3.1. Human Skin Samples

Human skin samples for the isolation of NHEK and NHDF (normal human dermal fibroblasts) were obtained from surgical excisions. Patients signed the informed consent approved by the Institutional Commission of Ethics of the University of Heidelberg (103/2001) and following the Declaration of Helsinki. Skin samples were derived from both male and female donors of different ages and included leg, trunk and facial skin.

### 3.2. Cell Culture

NHDF and NHEK were isolated as previously described [46]. NHEK were cultured at 37 °C, 5% CO_2_ and 20% O_2_ in low-Calcium, serum-free DermaLife K medium (Lifeline; Carlsbad, CA, USA) and NHDF at 37 °C, 5% CO_2_ and 5% O_2_ in high glucose and l-glutamine-containing Dulbecco’s modified Eagle’s medium (DMEM; Lonza, Verviers, Belgium). The DMEM was supplemented with 10% fetal calf serum (FCS; Invitrogen, Darmstadt, Germany) and 1% antibiotics (Lonza) Medium was generally changed every 2–3 days. For subculturing, NHEK were treated with 0.05% EDTA (Serva Electrophoresis GmbH, Heidelberg, Germany) for 5 min and 0.4% trypsin for 2 min, while NHDF were treated with 0.05% trypsin for 5 min only. Mycoplasma and virus contamination was excluded by the Multiplex Cell Contamination Test (Multiplexion) (Heidelberg, Germany).

HEK293 and A431 were a gift from Prof. Herrmann-Lerdon (DKFZ, Heidelberg, Germany), were cultivated in DMEM and passaged once per week.

Human induced pluripotent stem cells (hiPSCs) were produced and kindly provided by Prof. Jochen Utikal (DKFZ, Heidelberg, Germany).

### 3.3. Generation of Fibroblast-Derived Matrix-Based Skin Equivalents

The procedure to generate the skin equivalent is described in detail in the recent publication [10]. In brief, NHDF cells are seeded onto a filter insert in three successive steps in the course of one week. During a submerged cultivation period of four weeks, the fibroblasts create a dermal equivalent (DE) consisting of a dense extracellular matrix. NHEK are seeded onto the DE and subsequently cultivated at the air-liquid interface. The keratinocytes grow into a fully-stratified epidermis that can be maintained for up to six months.

### 3.4. Indirect Immunofluorescence

For staining of whole SE specimens (whole mounts), the tissue was fixed in 2% formaldehyde for 2 h, permeabilized in 0.2% Triton-X in phosphate-buffered saline (PBS) for 15 min and blocked in blocking buffer (5% donkey serum (Dianova, Hamburg, Germany), 5% goat serum (Dianova), 5% BSA in PBS) for 1 h. Primary antibodies were incubated over night at 4 °C in blocking buffer followed by fluorophore-conjugated secondary donkey antibodies and 2  μg/mL DAPI (Sigma-Aldrich, Taufkirchen, Germany) incubation for 2 h at RT. The whole mounts were mounted in Fluorescent Mounting Medium (DAKO) and images taken with the confocal Leica TCS SP5 II (Leica, Wetzlar, Germany). Samples were analyzed at 40× magnification. Images of 1024 × 1024 pixels with a pixel size of 0.4 μm were acquired. Z-stacks were imaged at intervals of 0.7 μm.

For cryosections, the skin equivalents were embedded in Tissue Tek O.C.T. and frozen in the gas phase of liquid nitrogen. Frozen specimens were cut into 6 µm sections at a cryotome and fixed 10 min in 4% Formaldehyde, permeabilised 5 min in 0.2% Triton-X in phosphate-buffered saline (PBS), and blocked 30 min with blocking buffer. Antibody incubation was done as for the wholemounts, with incubation times over night and 1 h, respectively. The sections were mounted in Fluorescent Mounting Medium (Dako) and images were performed using an Olympus Provis AX70 fluorescence microscope (Olympus, Hamburg, Germany) that is equipped with a F-View CCD camera and controlled by Cell^F software.

Primary antibodies used were: anti-H3S10ph (Epitomics, Burlingame, CA, USA, 1173-1), anti-Keratin K10 (Progen, Darra, QLD, Australia, 11,414), anti-Integrin α6 (Progen 10,709), anti-Numb (peptide against aa 537–551; courtesy of S. Pece, Milan, Italy; see [47]), anti-Ki67 (Abcam, ab15580, Cambridge, UK). Secondary antibodies: donkey-anti-rabbit-DyLight488 (Invitrogen A21206), goat-anti-mouse-Cy3 (Jackson, West Grove, PA, USA, 115-165-068), goat-anti-rat-Alexa488 (Jackson 112-545-003).

### 3.5. Histological Processing

The tissue was fixed in 3.8% formaldehyde for at least 24 h before embedding in paraffin and cutting. The paraffin sections were freed from paraffin with xylol for 8 min. The sections then underwent an alcohol series of decreasing concentration (96%, 80%, 70%, 60%, 4 min each) before incubating for 2 min in ddH_2_O. The staining was done in a two-step process. First, the sections were incubated in the hematoxylin solution for 6 min and washed for 8 min under running tap water, followed by 6 min of staining in a 1% eosin solution and washing for 6 min. Finally, the sections were dehydrated by an increasing alcohol series (1 min 80%, 2 × 2 min 96% ethanol), 2 min in isopropanol and 2 × 5 min in xylol and embedded with EukittR.

### 3.6. CRISPR/Cas9 Knockdown

Five different guide RNA (gRNA) sequences (Table 1) were designed using the software designed by Hsu and colleagues [48], found at http://crispr.mit.edu/. The guide sequences were complimentary to the first exon, shortly after the start codon, and present in all four Numb isoforms found in skin, but not the Numb-like homologue.

**Table 1:** CRISPR guide RNA sequences. Five different guide RNAs were designed complimentary to the Numb gene sequence and with minimal off-target binding.

CRISPR gRNA Name	gRNA Sequence	Spacer Sequence
#1	GATGAAGAAGGCGTTCGCACCGG	GATGAAGAAGGCGTTCGCAC
#2	CTGCCACTGATGTGGACGACTGG	CTGCCACTGATGTGGACGAC
#3	TGATGTGGACGACTGGCCTCTGG	TGATGTGGACGACTGGCCTC
#4	AGGCCAGTCGTCCACATCAGTGG	AGGCCAGTCGTCCACATCAG
#5	CACCGGAAAATGTAGCTTCCCGG	CACCGGAAAATGTAGCTTCC

These sequences were cloned into the pSpCas9(BB)-2A-GFP (PX458) plasmid, additionally encoding the Cas9 sequence, as well as a GFP protein as a transfection marker. pSpCas9(BB)-2A-GFP (PX458) was a gift from Feng Zhang (Addgene Plasmid # 48138) [49].

The functionality of the plasmids was checked in HEK293 cultures. A T7 endonuclease test proved efficient gene cleavage. In brief, during the T7 test, the gene region around the double strand break is PCR amplified. The PCR products are heat denatured and reannealed to create heteroduplexes. These are recognized and cut by the T7 endonuclease. As a read out, the fragments are separated on a gel. The highest band indicates the wildtype gene region, while two smaller double bands are the cleaved fragments and, thereby, show effective gene cleavage by Cas9.

### 3.7. Electroporation

For transfection of the keratinocytes, the Invitrogen NEON™ electroporation system was used. Freshly-thawed keratinocytes were cultivated in DermaLife for 5 days up to a confluency of 70%–80%. The cells were trypsinized, counted and taken up in DermaLife without supplements. Plasmid DNA in an end concentration of 10 µg/mL was added, and 2.5 × 10^5^ cells were taken up in 100 µL with the NEON™ transfection pipet and the appropriate transfection tip. After pulsing the cells once for 40 ms at 1100 V, they were directly transferred into collagen-coated 6-well plates containing pre-warmed DermaLife and cultivated for 48 h.

### 3.8. Fluorescence-Activated Cell Sorting

Cells were trypsinized, and approximately 2 × 10^6^ cells per 1 mL were taken up in PBS with 2% FCS with 1 µL 7AAD for live staining of the cells. Cells were sorted at the DKFZ core facility and collected in 5 mL Falcon tubes containing medium. The collected cells were processed immediately after sorting.

### 3.9. Protein Detection by Western Blot

Cells were lysed with RIPA buffer for 30 min on ice. After centrifugation for 30 min at 14,000 rpm, the protein content of the supernatant was measured with a BCA assay. The proteins were separated on a 10% SDS gel and blotted onto a nitrocellulose membrane. After blocking the membrane for 1 h in 5% skim milk in 0.1% PBS-T, proteins were detected with primary antibody in PBS-T over night at 4 °C and HRP-coupled secondary antibody in blocking milk for 1 h at RT with subsequent luminometric detection with ECL (GE, Buckinghamshire, UK).

### 3.10. Microscopy

For whole mount microscopy, the confocal Leica TCS SP5 II was used. Samples were analyzed at 40× magnification. Images of 1024 × 1024 pixels with a pixel size of 0.4 µm were acquired. z-stacks were imaged at intervals of 0.7 µm.

For live imaging of keratinocytes in 24-well plates, an Olympus IX81 inverted fluorescence microscope (Olympus, Hamburg, Germany) with an incubation chamber was used. The plates were automatically imaged every 10 min at 20× magnification.

## Supplementary Materials

Supplementary materials can be found at http://www.mdpi.com/1422-0067/17/2/167/s1.

## Abbreviations

BM: Basement Membrane
FACS: Fluorescence-activated cell sorting
IFE: Interfollicular epidermis
K10: Keratin K10
NHEK: Normal human epidermal keratinocytes
NICD: Notch intracellular domain
SE: Skin equivalent
